# Enhanced band-edge photoluminescence from ZnO-passivated ZnO nanoflowers by atomic layer deposition

**DOI:** 10.1186/1556-276X-8-105

**Published:** 2013-02-25

**Authors:** Zhi Wei Ai, Yun Wu, Hao Wu, Ti Wang, Chao Chen, Yang Xu, Chang Liu

**Affiliations:** 1 Key Laboratory of Artificial Micro- and Nano-structures of Ministry of Education, School of Physics and Technology, Wuhan University, Wuhan 430072, People’s Republic of China

**Keywords:** ZnO nanoflowers, Atomic layer deposition, Photoluminescence, 77.55.hf, 73.63.Bd, 73.40.Kp

## Abstract

The ZnO nanoflowers were synthesized by reactive vapor deposition. A secondary nucleation in the stalk/leaves interface was suggested. The photoluminescence revealed that there were many oxygen vacancies in the nanoflowers. To tune the optical properties of ZnO nanoflowers, ZnO thin films with varying thicknesses were coated on the nanoflowers by atomic layer deposition, which can distinctly improve the band-edge photoluminescence properties.

## Background

Recently, one-dimensional (1-D) zinc oxide nanostructures including nanocages [1], nanotubes [2], cylindrical nanowires [3], nanorods [4], nanoribbons, and belt-like nanostructures have been obtained. Zinc oxide nanostructures attracted much attention due to their wide direct band gap of 3.37 eV and large exciton binding energy of 60 meV at room temperature [5-12] and their great potential applications in solar cells [13], piezoelectric devices [14], gas sensors [15], and UV laser diodes [16]. ZnO nanostructures can be synthesized by reactive vapor deposition under controlled conditions. By changing the growth conditions, different ZnO nanostructures have been prepared. On the other hand, atomic layer deposition (ALD) is good at control of the accuracy, homogeneity, consistency, and thickness of the thin coatings, which brings it to be a good way for the surface modification and enhancement.

In this work, we demonstrate the growth of ZnO nanoflowers by reactive vapor deposition. To improve the optical properties, the ZnO thin films with varied thicknesses from 15 to 45 nm were coated on the nanoflowers by ALD. This thin-coated layer does not change the morphologies of the sample but can greatly improve its optical properties.

## Methods

The growth of ZnO nanostructures was performed in a horizontal tube furnace. Zn powder (99.9%) with a weight of 1 g was loaded in quartz boat and placed into the center of the tube furnace, and the clean Si substrates were located at 2 cm downstream from the Zn source. Afterwards, the tube furnace was heated to 440°C with a rate of 20°C/min and held there for 60 min. During the whole synthesis process, a constant flow of O_2_/Ar mixed gas (5%) at 30 sccm was introduced into the system and the pressure in the tube was kept about 200 Pa.

The as-grown ZnO nanoflowers were coated with thin ZnO layers grown by ALD with a TSF-200 machine (Beneq Oy, Vantaa, Finland). Diethyl zinc (DEZn) and deionized water (H_2_O) were used as the sources of zinc and oxygen, respectively. High-purity nitrogen carrier gas was used to load DEZn and H_2_O to the chamber and cleanse the redundant former precursor. The temperature of the substrate was held at 200°C. In each identical ALD cycles, DEZn was introduced into the chamber firstly for 0.2 s, and afterward the chamber was purged by N_2_ for 1 s. In succession, H_2_O was introduced into the chamber for 0.2 s followed by another purging procedure at 1 s. The thickness of the ZnO film was about 15 nm after 100 cycles were performed.

X-ray diffraction (XRD; Bruker D8 Advance, Bruker AXS GmbH, Karlsruhe, Germany) and high-resolution transmission electron microscopy (HRTEM, JEOL JEM 2010 FEF UHR; JEOL Ltd., Tokyo, Japan) were used to analyze the crystallization and the microstructure of the ZnO nanoflowers. The morphologies of the sample were characterized by a Sirion (FEI Company, OR, USA) FEG scanning electron microscope (SEM). The photoluminescence (PL, Horiba LabRAM HR800; HORIBA Jobin Yvon S.A.S., Longjumeau, Cedex, France) spectra were utilized at room temperature in a wavelength range of 350 to 700 nm to analyze the optical properties of the ZnO nanoflowers and the coated films.

## Results and discussion

**Figure 1:**
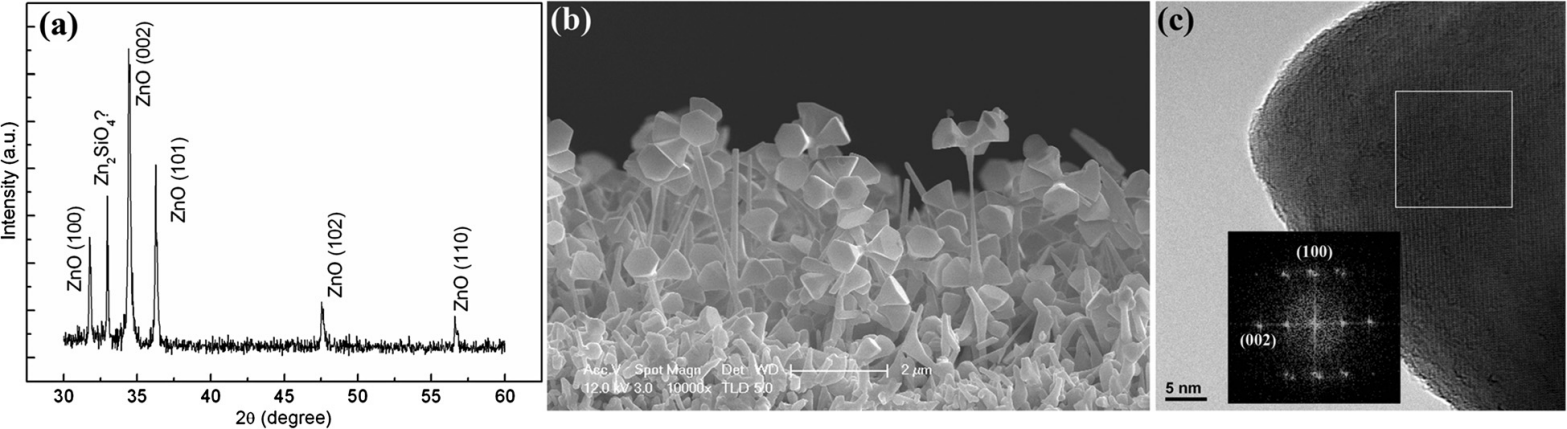
**XRD diffraction pattern and side-view SEM and HRTEM images of ZnO nanoflowers. **(**a**) XRD diffraction pattern of the as-grown ZnO nanoflowers; (**b**) the side-view SEM image of the as-grown sample, showing that the ZnO is a flower-like; (**c**) HRTEM image of the stalk of the nanoflowers. The inset (**c**) shows the DDPs of the marked region.

Figure 1a shows the XRD patterns of the as-grown ZnO nanoflowers. The diffraction peaks of ZnO can be observed. An additional peak located at 33.40° possibly comes from Zn_2_SiO_4_ (112) (JCPDS 24–1467), which may be formed due to the zinc diffusing into the Si/SiO_2_ substrate during the growth.

**Figure 2:**
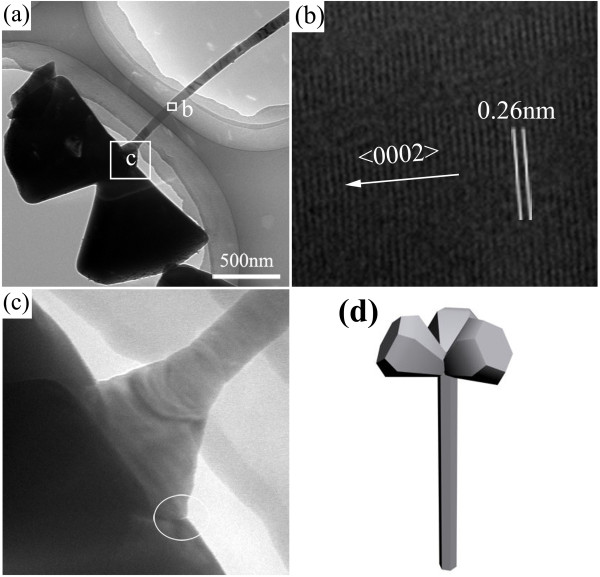
**TEM and HRTEM images and sketch map of the structure of one ZnO nanoflower. **(**a**) TEM image of one ZnO nanoflower; (**b**) HRTEM image of the region in the stalk, which is marked by a small square in (**a**); (**c**) the enlarged image corresponding to the region marked by the big square in (**a**); (**d**) a sketch map of the nanoflower structure.

Figure 1b shows the side-view SEM image of the as-grown sample. The image shows that ZnO grew firstly to form a thin ‘stalk’ with a length of about 3 μm and a shrinking diameter from about 50 to 100 nm, then grew radially outward on the top of the stalk to form several hexagonal ‘leaves’, which can be more distinctly observed in Figure 2a,b. The leaves are like the hexagonal wimble with the shrinking diameter from 500 nm (on the top) to 100 nm (where connected with the stalk). This structure is similar with that reported by Lao et al*.*[10]. Figure 1c shows the HRTEM image of one ZnO stalk. It is single crystalline. The digital diffraction patterns (DDPs) obtained by fast Fourier transformation of the marker region is shown in the inset, indexed and determined the wurtzite structure of ZnO orientations. The direction of the stalk is along the (0002) orientation of ZnO.

The nanowires in the junction of the branch are not very smooth. Therefore, we suggest that this branching should not be the epitaxy of a nanowire crystal face. It belongs to the secondary nucleation phenomena, which means that the nanowires grow to a certain length along the *c*-axis and a secondary nucleation appears at the top the nanowire. These crystal nuclei grow along the new nuclear *c*-axis and form a flower-like structure. To verify our hypothesis, the samples were analyzed by transmission electron microscopy (TEM) in the following text.

Figure 2a shows the TEM image of the nanoflower structure of the nanowires. Figure 2b shows the HRTEM image of the region marked by the white square b in Figure 2a, which is located in the stalk. The interplanar distance of 0.26 nm is corresponding to the wurtzite ZnO (0002) planes; hence, the growth orientation is along the *c*-axis. Figure 2c is the enlarged image corresponding to the region marked by the white square c in Figure 2a. A gap can be observed at the joint parts between the stalk and leaves, which is marked by the white circle in Figure 2c. This suggests that the leaves structure does not belong to the same epitaxial structure of the stalk, but rather due to the secondary nucleation. The growth mechanism of the nanoflower structure can be described as below: First, the nanowire grows along *c*-axis direction with a wurtzite structure. Then in the top region of the nanowire, there is secondary nucleation, and the *c*-axis of the new ZnO grains deviates from the direction before. The end planes of the leaves structures show the regular hexagon. These branches exhibit symmetry due to the constraints from space position.

**Figure 3:**
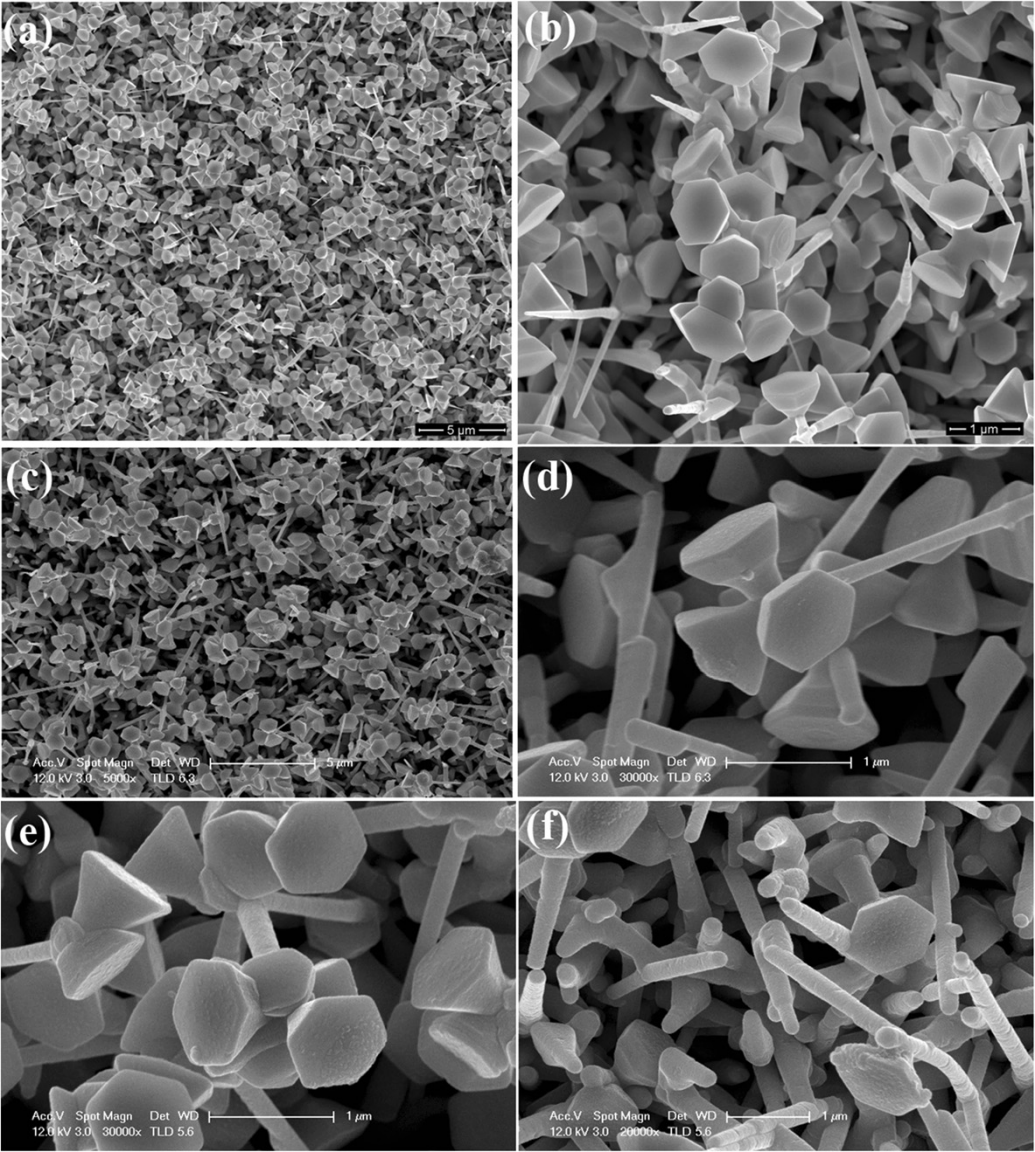
**SEM images of different samples with varying magnifications. **(**a,b**) The as-grown ZnO nanoflowers; (**c,d**) the nanoflowers coated by a ZnO thin film with a thickness of 15 nm by ALD; (**e,f**) the nanoflowers coated by the ZnO thin films with the thicknesses of 30 and 45 nm, respectively.

Figure 3 shows the top-viewed SEM images of the as-grown nanoflowers and the coated sample. The hexagonal leaves and the thin stalk can be observed. Since the ZnO thin film grown by ALD is only 15 ~ 45 nm thick, the morphologies of the as-grown sample and the sample coated by ZnO film are nearly the same, revealing the good conformality of the coating film.

**Figure 4:**
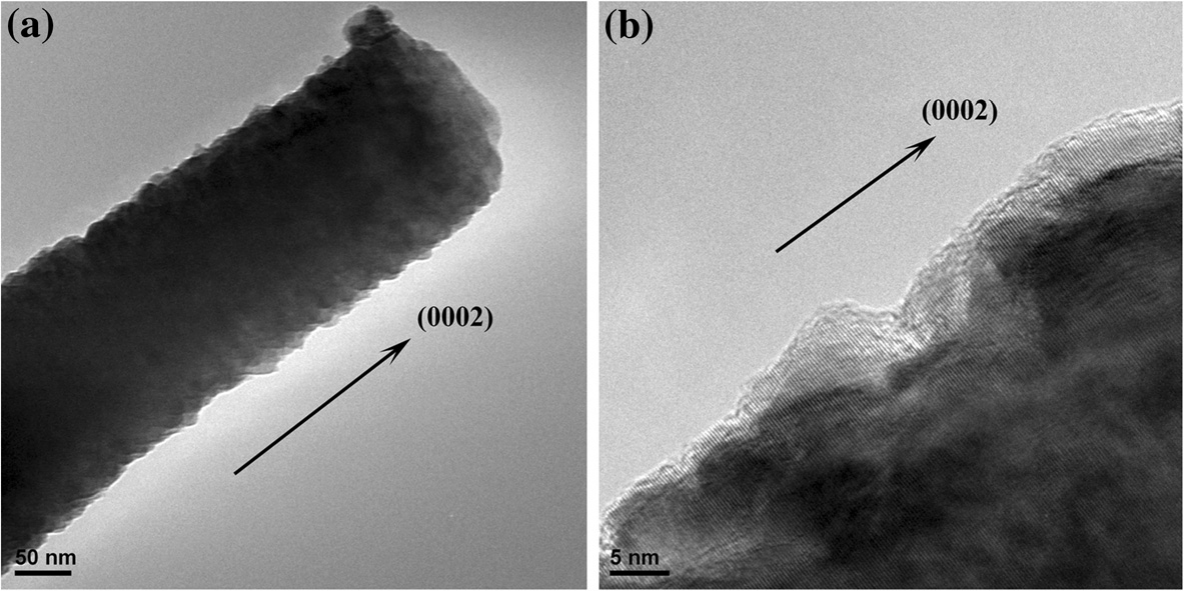
**TEM and HRTEM images of the ZnO stalk coated by a ZnO thin film. **TEM image of the ZnO stalk coated by a ZnO thin film with a thickness of 15 nm by ALD (**a**) and the HRTEM image of this sample (**b**). The layered structure can be observed in the sideward regions of the ZnO stalk, which is corresponding to the coated ZnO films.

Figure 4 shows the TEM images of the ZnO stalk coated with 15-nm ZnO thin film. As shown in the HRTEM image in Figure 4b, the sideward regions of the ZnO stalk show a distinct layered structure, which can be attributed to the coated ZnO thin film, implying that the coated thin film is also crystalline and its orientation is the same as the as-grown ZnO nanoflowers. From this image we can suggest that the coated ZnO thin films by ALD are epitaxial. There are some amorphous regions with the thickness of several angstroms at the boundary, which may be due to the electron beams in the process of the TEM.

**Figure 5:**
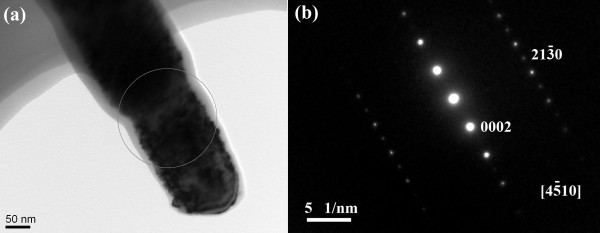
**TEM image of a ZnO stalk and corresponding SAED image. **The TEM image of a ZnO stalk coated with 45-nm ZnO thin film (**a**) and the corresponding SAED image (**b**). Only one set of lattice due to the ZnO can be observed.

To confirm that our ZnO thin films are epitaxial, we performed the selected area electron diffraction (SAED) measurement of our samples. The TEM image of the ZnO stalk coated with 45-nm ZnO thin films is shown in Figure 5a, and the corresponding SAED image is shown in Figure 5b. From the SAED image, it can be concluded that the ZnO stalk is grown along *c*-axis. Moreover, there is only one set of diffraction lattice, which is attributed to ZnO. Hence, we can claim that our coated ZnO thin film is epitaxial; otherwise, there will be another diffraction spots or rings.

**Figure 6:**
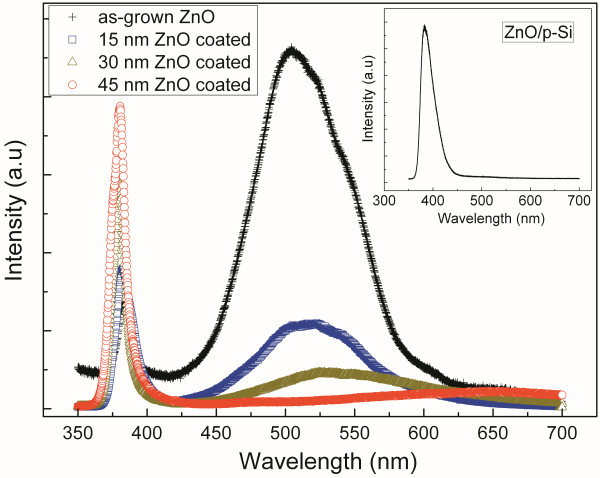
**Room-temperature PL spectrum of the as-grown and ZnO-passivated ZnO nanoflowers. **The room-temperature PL spectrum of the as-grown ZnO nanoflowers and the samples coated by the ZnO thin films with varied thicknesses. The inset shows the PL spectra of the ZnO thin film by ALD on silicon substrate.

The room-temperature PL spectra of the as-grown and coated samples are presented in Figure 6. As shown, the spectrum of as-grown ZnO nanoflowers (the black crosses) displays a dominant deep level emission around 520 nm, contrasting to a weak band-edge transition peak around 380 nm. It is well known that the deep-level emissions were from zinc interstitials or oxygen vacancies. According to our preparation method of the ZnO nanoflowers, the most possible defects may be that zinc cannot be oxidized sufficiently, which will induce the oxygen vacancies or zinc interstitials, leading to a strong deep-level emissions. The ratio of the intensity of the band-edge transition to that of the deep-level emissions is used to reveal the effect from the deep-level emissions. For the as-grown sample, this ratio *α* is about 0.28.

To improve the optical properties, the as-grown sample was coated by a ZnO thin film by ALD. It was shown that ZnO films grown by ALD would have few zinc interstitials and oxygen vacancies [17]; hence, it is a good way to improve the optical properties of the nanostructures. After a ZnO film was coated, with thickness about 15 nm (the blue squares), the deep-level emission decreased dramatically about 80%; moreover, the intensity of band-edge transition increased about 30%. The ratio *α* is about 1.65. This result reveals that the very thin film on the surface of the nanoflowers can effectively enhance their optical properties without altering the morphologies.

With the increasing thickness in the coating of ZnO films, the deep-level emission decreases and the band-edge transition increases, as shown in Figure 6. The deep-level emission of the sample coated with 45 nm ZnO is only 4% of that from the as-grown sample. In addition, the intensity of the band-edge transition from the sample coated with 45-nm ZnO is 300% more than that from the as-grown sample. The ratios of the intensity of the band-edge transition to the deep-level emissions are 5.91 and 16.5 for the samples with 30-nm and 45-nm ZnO, respectively.

These results show that an ALD coating of ZnO thin films can effectively enhance the optical properties of the ZnO nanostructures. However, we should know whether the PL result is due to the original ZnO nanoflower or from the ALD ZnO. Hence, we fabricated the ZnO thin film on silicon substrate by ALD using the same parameters. The thickness of this ZnO film is 45 nm, and the PL spectrum of this sample is shown as the inset of Figure 6. A strong peak around 382 nm can be observed, which is attributed to the band-edge transition. Moreover, there is nearly no deep-level emission in the sample. Hence, we can make a conclusion that the stronger peak of the band-edge transition is mostly from the ZnO thin films by ALD, while the weaker peak of the deep-level emission is from the original ZnO nanoflowers. Usually, in the ZnO nanostructures, there are many oxygen vacancies and zinc interstitials, so their optical properties are very poor. Our result reveals that we could coat an epitaxial ZnO thin film by ALD on these nanostructures. This method can effectively enhance the optical properties without changing the morphologies.

Another point should be noted that there is a blue shift in the band-edge transitions and a red shift in the deep-level emissions with increasing the thickness of the coating ZnO films. This reason needs further investigation.

## Conclusions

In conclusion, we have synthesized ZnO nanoflowers by reactive vapor deposition. The ZnO thin films were coated on the pristine nanostructures by ALD. These thin-coated layers could remarkably improve the UV band-edge photoluminescence of the nanoflowers without changing their morphologies. Our method can provide an effective way to enhance the performance of the possible ZnO nanostructure devices.

## Competing interests

The authors declare that they have no competing interests.

## Authors’ contributions

ZWA fabricated the ZnO thin films, performed the measurements of the TEM, and wrote the manuscript. YW grew the ZnO nanoflowers. HW analyzed the results, performed the measurements of the SEM, and wrote the manuscript. TW helped to measure the PL spectra. CC helped to grow ZnO films. YX helped in the TEM measurement. CL supervised the overall study. All authors read and approved the final manuscript.
